# Dynamics of price formation on complex networks

**DOI:** 10.1093/pnasnexus/pgaf014

**Published:** 2025-01-31

**Authors:** Andrea Civilini, Vito Latora

**Affiliations:** School of Mathematical Sciences, Queen Mary University of London, London E1 4NS, United Kingdom; Dipartimento di Fisica ed Astronomia, Università di Catania and INFN, Catania I-95123, Italy; School of Mathematical Sciences, Queen Mary University of London, London E1 4NS, United Kingdom; Dipartimento di Fisica ed Astronomia, Università di Catania and INFN, Catania I-95123, Italy; Complexity Science Hub Vienna, Vienna A-1080, Austria

**Keywords:** econophysics, price formation, competition, complex networks

## Abstract

Networks mediate our economic interactions. From spatial networks representing transportation routes, to more abstract networks of similarity among products, we interact, trade, and compete over networks with a complex structure. Nevertheless, classical economic models of how prices are formed under competition, such as the Edgeworth and the Hotelling models, completely neglect the network structure of real markets. Here, we introduce a dynamical model of price formation, where sellers and buyers are placed on the nodes of a network with any arbitrarily complex topology. Buyers are modeled as random walkers with perfect or limited information about the market, and their distribution depends on the prices and positions of sellers. Concurrently, sellers dynamically adjust their prices to maximize their payoff, based on the current distribution of buyers. These two dynamical processes coevolve and we show that, depending on the positions of the sellers and on the level of information available to the buyers, the model can either converge to fixed prices, or produce cycles of different amplitudes and periods, similar to those observed in real-world markets. By unveiling how competition depends dynamically on the structure of a market, our model allows to quantify the strength of competition in real-world markets and also to identify the most profitable locations for a seller. We use urban street patterns of different cities from all over the world, as examples of spatial networks, to illustrate a novel measure of node centrality, which ranks nodes based on the payoff earned by a seller placed on that node.

**Significance Statement**

Many real-world markets are oligopolies, economic systems where prices are shaped by competition among a small number of entrepreneurs. Despite price formation in oligopoly models having been studied since the end of the 19th century, understanding how and why different price patterns emerge remains an open question. In particular, both stable and cycling prices are observed in real markets. Here, we introduce a model of price formation where structured markets are represented as networks. Depending on network topology, seller positions, and information levels, our model produces both fixed prices and cycles with regular and irregular periods in a unified framework. This also opens the possibility of using price dynamics to identify the most profitable markets and positions for a seller.

## Introduction

Oligopoly pricing, i.e. how market prices are formed in the presence of two or more competitors, is a central topic in economic theory. The origin of the interest in oligopoly pricing can be tracked back to a series of classical papers from the end of the 19th century, which can be regarded as the ancestors of modern noncooperative game theory (1, 2). In 1883, Bertrand showed that, in the presence of price competition, the Cournot model of duopoly (3) led to the famous Bertrand’s Paradox, unrealistically predicting perfect competition (i.e. zero profit) even in the simple case of two sellers (4). A first solution to the Bertrand’s paradox was proposed by Edgeworth in 1925: by introducing sellers with capacity constraint, i.e. such that each one of the sellers alone is unable to serve the whole market, he showed the emergence of the so-called Edgeworth price cycles, oscillations in the prices having positive profit for the sellers (5). In his seminal 1929 work, Hotelling first noticed how the market structure can have a great impact on the stability of prices in an oligopoly (6). He introduced a model where two sellers occupy different positions on a market described as a line segment. The line here represents a simplified spatial market, e.g. the main street in a town, where the sellers are placed along the street and are separated by geographical distance. However, the market structure is not limited to representing the geography of a system. As noticed by Hotelling himself, the line can also be interpreted as a measure of a given feature/quality of a product (e.g. the different levels of sourness/sweetness of a cider, or the different colors of a pair of trousers). In such a case, the similarity of two products is defined by the distance of their positions on the line. Hotelling showed how in his model prices converge to nontrivial fixed values with positive profit as a consequence of the market structure and of the positions occupied by the sellers.

Even if qualitatively opposite conclusions can be drawn from the solutions proposed by Edgeworth and Hotelling to the Bertrand’s paradox, both fixed (à la Hotelling) and cycling (à la Edgeworth) prices with positive profit have indeed been observed in the real world. In particular, thanks to the increasing amount of fine-grained prices’ data available, Edgeworth price cycles, once thought a purely theoretical construction (7), have been recently observed in real-world markets such as the retail gasoline market and online bid platforms (8, 9). Despite these fundamental results, a full understanding of the link between price dynamics and the structure of a market is still missing. Firstly, due to its mathematical complexity, a general solution of the Hotelling model on a line segment has not yet been found (10–16). Moreover, a line segment is a too strong and unrealistic assumption (10, 11), and this greatly limits the applicability of the Hotelling model to complex real-world markets (17–20). Finally, it is also challenging to incorporate in the Hotelling model realistic aspects of the decision making process, such as bounded rationality and cost of information (21, 22), which have been proved to play a central role in many complex systems (23–27).

Here, we propose a dynamical model of price competition in which sellers and buyers are placed on the nodes of a graph of any arbitrary topology. Given their great flexibility in representing interactions in complex systems (28–31), graphs are the natural choice for modeling real-world markets (32–39). For instance, they can better capture the geographical aspects of a market (40, 41) than a simple line segment. They are also better suited to describe the complex pattern of relations among a set of products, given that the similarity/difference between two products is often based on the degree of overlap of more than a single feature (42, 43). In our model of price competition the distribution of buyers over the graph is not fixed, but dynamically depends on the prices and positions of sellers. At the same time, the price dynamics is influenced by the distribution of the buyers. In this way, our model allows to include different price update rules and also buyers with limited information about the market (bounded rationality), by naturally modeling them as random walkers.

We find that the model exhibits a very rich dynamics. In fact, depending on the positions of the sellers on the graph, and on the level of information available, the prices can either converge to fixed values (à la Hotelling), or produce Edgeworth cycles of different amplitudes and periods. As an additional benefit, the price dynamics of the model allows us to define a set of metrics to characterize the structure of a real-world market, both at a global and at a local scale. First, we show that the maximum prices scale with the size of a market. By extracting the scaling exponent, we can then define the “market competition dimension” of a network. This is a quantity that allows to measure, at a global scale, the level of competition of sellers in a given structured market. As an application, we find that the street networks of real-world cities considerably enhance competition compared to artificial graphs such as regular square lattices. Then, at a microscopic scale, we use the average payoff as a function of the seller position to define a novel measure of node centrality that we name “Hotelling centrality” (HC). Such a centrality identifies and ranks the more profitable positions on a market as a function of the level of information of the sellers.

## Model framework

In our model, the market units are the nodes of an undirected graph (28), whose links can represent either geographical adjacency or feature similarity. The structure of the graph can be arbitrarily complex and is described by the adjacency matrix $A=\{a_{ij}\}$, whose element $a_{ij}$ is equal 1 if node *i* and *j* are connected by an edge, otherwise is 0. Sellers and buyers are placed on the nodes of the graph. We focus on the case of two sellers, *α* and *β*, located at nodes $n_{\alpha }$ and $n_{\beta }$, selling a commodity at prices $p_{\alpha }$ and $p_{\beta }$, although the model can be readily extended to include additional sellers. The process by which buyers search for the seller to buy from is modeled as a random walk with a tunable level of rationality, *w*. That is, with probability 1−w, a buyer has bounded rationality and moves as a lazy random walker. This means that a buyer at node *i* either jumps to one of the neighbors of *i* on the graph with uniform probability or it remains on node *i*, as long as the price difference $\left|p_{\alpha }-p_{\beta }\right|$ is below a given threshold $\Delta p_{T}$ (representing the limit of bounded rationality). Instead, with a probability *w*, the buyer at node *i* is fully informed about the positions and prices of the sellers. In this case, at each time step, it moves on the graph towards the seller *α* with the lower delivered price:


(1) $$P_{i\alpha }=p_{\alpha }+d_{i\alpha }.$$

The delivered price $P_{i\alpha }$ combines the actual price $p_{\alpha }$ of the product sold by seller *α* together with the graph distance $d_{i\alpha }$ from node *i* to the node occupied by the seller *α* (for further discussion on buyers’ dynamics, see Supplementary material). This tradeoff between rational and random search is in line with existing models of buyers–sellers dynamics in different contexts. For example, in Ref. (34), the authors model a real-world fish market, where buyers are connected to sellers by loyalty bounds (i.e. a measure of social distance) and they can change these bounds based on prices and random exploration. In spatial networks, the distance can instead be directly interpreted as the transportation cost to reach the seller. Instead, on a network of feature similarity, the position occupied by sellers represents the features of the product sold by the seller, while the position of a buyer reflects its ideal preference. Therefore, in this case, $d_{i\alpha }$ measures the distance between the preference of a buyer at node *i* and the offer of seller *α*. The row vector $\phi (t)=\{\phi_{i}(t){\}}_{i=1,\ldots ,N}$, representing the distribution of buyers across the *N* nodes of the network at time *t*, evolves over time according to ϕ(t)=ϕ(t−1)Π. Here, *Π* is the transition matrix governing the dynamics of random walkers on the network, where each element $\Pi_{ij}$ denotes the probability of a buyer transitioning from node *i* to node *j* (see Materials and methods for further details). An important assumption in our model, which is consistent with the original Hotelling model, is that prices evolve on a slower time scale than the buyer dynamics. That is, we assume that prices do not change in the time between the evaluation of the lowest price and the purchase of the good, similarly to what happens in many real-world markets (e.g. in a mall or in a city’s main street), where buyers plan their visit to a shop attracted by price and the price remain stable on a daily or weekly basis. Once the buyers have reached their stationary distribution $\phi^{*}$ (i.e. a distribution that no longer evolves over time, such that $\phi^{*}=\phi^{*}\Pi$), the two sellers evaluate their payoffs. A seller payoff is calculated as the product of the seller price times the fraction of buyers visiting the node occupied by the seller: $\pi_{\alpha }=\phi_{\alpha }^{*}p_{\alpha }$ for seller *α* and $\pi_{\beta }=\phi_{\beta }^{*}p_{\beta }$ for seller *β*. A randomly selected seller, let us say *α*, can then change its price $p_{\alpha }$ in order to increase its payoff. We considered two different price update rules, the so-called One-Step (OS) and Best Response (BR). In the OS dynamics the seller slowly adjusts its price in steps of $\Delta_{p}=\pm 1$. Conversely, in the BR dynamics the seller can choose the new $p_{\alpha }$ from the set of prices that maximize its payoff, given the current price of the other seller. This set represents the best response to the competitor’s price, evaluated over the entire range of possible prices, i.e. without any restriction on the price step Δp.^a^ A new stationary distribution of buyers is then recalculated according to the new value of $p_{\alpha }$, i.e. the new delivered prices in Eq. 1. If the payoff associated with the new $p_{\alpha }$ is higher than the old payoff, the seller will adopt the new price, otherwise the seller will keep the old one. Hence, the price dynamics is driven by two opposite forces: while on the one hand increasing (decreasing) the price $p_{\alpha }$ will lead seller *α* to earn more (less) from each buyer, on the other hand it will reduce (increase) the number of sellers buying from seller *α*, as it causes an increase (decrease) of the delivered price $P_{i\alpha }$.

## Results

### Price dynamics

**Fig. 1.**
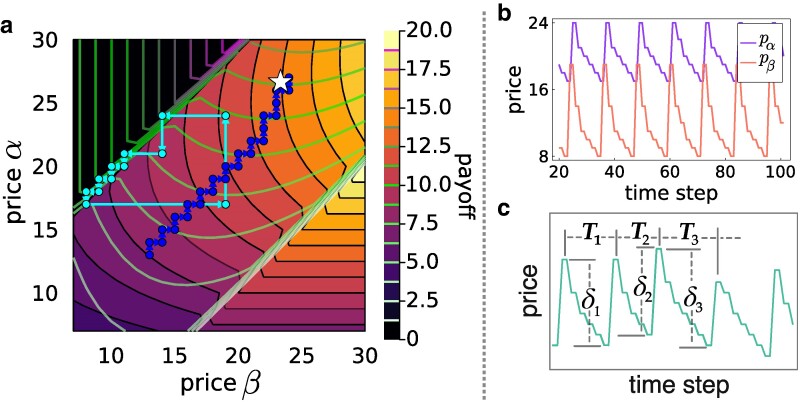
Price dynamics on a chain of N=25 nodes, with seller positions $n_{\alpha }=10$ and $n_{\beta }=20$. a) The heatmap shows the payoff of seller *α*, the payoff’s level curves of seller *β* are represented in green-magenta. The blue and cyan trajectories show the price dynamics under a OS and BR update, respectively. The OS converges to the equilibrium prices predicted by the continuous Hotelling linear model (white star), while the cyan trajectory shows price cycles. b) Time trajectories of the two players’ prices, showing the typical asymmetric patterns of the Edgeworth cycles. c) Sketch of the two quantities measured to characterize each cycle *i*, namely the period $T_{i}$ and the amplitude $\delta_{i}$.

**Fig. 2.**
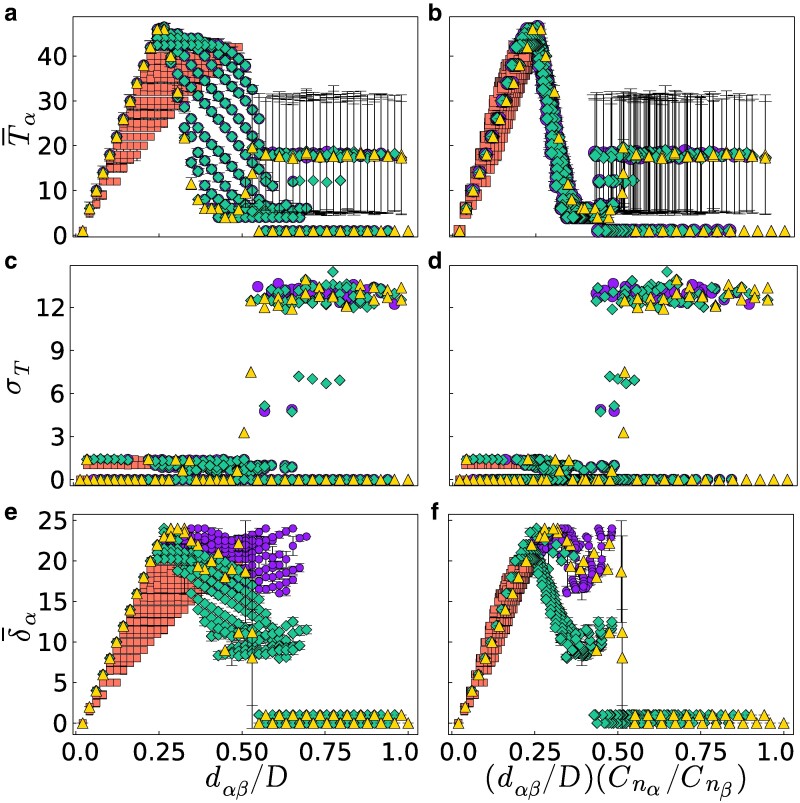
a,b) Average period $\bar{T}$ and c,d) its variance $\sigma_{T}$, and e,f) average amplitude $\bar{\delta }$ of the BR dynamics on a chain of N=50 nodes as a function of the normalized seller distances $d_{\alpha \beta }^{\prime }$ (left panels) and $d_{\alpha \beta }^{\prime \prime }$ (right panels). Denoting the chain’s middle point $x_{c}=25.5$ and the sellers center of mass $n_{c}=(n_{\alpha }+n_{\beta })/2$, the orange squares are for positions $\left(n_{\alpha },n_{\beta }\right)$ such that $n_{\alpha },n_{\beta }<x_{c}$, the purple circles, green diamonds, and yellow triangles are for $n_{\alpha }<x_{c}<n_{\beta }$ with $n_{c}<x_{c}-0.5$, $n_{c}>x_{c}+0.5$ and $|n_{c}-x_{c}|\leq 0.5$, respectively. The vertical bars are the SD.

**Fig. 3.**
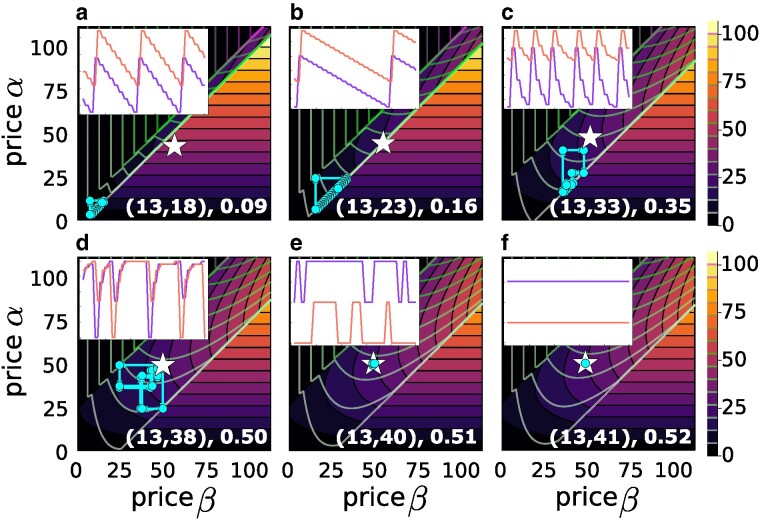
Price trajectories for increasing seller distances. The values $\left(n_{\alpha },n_{\beta }\right)$, $d^{\prime \prime }$ are shown in the bottom-right of each panel. For $d^{\prime \prime }<0.5$, Edgeworth cycles appear: a, b) $\bar{T}$ and $\bar{\delta }$ increase with distance for $d^{\prime \prime }<0.25$, then c) decrease for $0.25<d^{\prime \prime }<0.5$. d) Irregular reverse cycles occur near $d^{\prime \prime }=0.5$. e, f) For $d^{\prime \prime }>0.5$, either Hotelling fixed points or irregular oscillations around them are observed.

We first characterize the price dynamics of our model in the simplest case in which the sellers are placed on a chain of *N* nodes and the buyers have perfect information, i.e. w=1. Consistently with the original formulation by Hotelling, we assume here a uniform initial distribution of the buyers over the nodes, which can represent their residence in a spatial network as a city. For w=1, each buyer moves towards the seller with the lowest delivered price with probability 1. This effectively assigns each buyer *i* to the seller *α* with the lowest $P_{i\alpha }$ in a deterministic manner, analogously to the original Hotelling model. Notice that a chain is a generalization of the line segment originally used in the Hotelling model, as for large *N* the chain reduces to the line segment. Such a simple topology is still of theoretical interest. In fact, the equilibrium prices initially found by Hotelling (6) have been subsequently proven to be correct only when the two sellers are *not too close*. Therefore, the Hotelling model on a line segment still lacks a general solution (10–16). Instead, with our approach, we will not only be able to provide a complete characterization of the solutions but also to explore the effect of different price update dynamics and levels of information. When the sellers occupy two nodes $n_{\alpha }$ and $n_{\beta }$ at distance $d_{\alpha \beta }$ on a chain, we expect to recover the equilibrium prices corresponding to the Nash Equilibrium (44) of the original Hotelling model (6):


(2) $$p_{\alpha }^{H}=N+\frac{a-b}{3}$$

(3) $$p_{\beta }^{H}=N-\frac{a-b}{3},$$

when *N* and $d_{\alpha \beta }$ are sufficiently larger than 1 and in the case of perfect rationality w=1. Here, *a* and *b* are the distances of sellers *α* and *β*, respectively, from the closer extremity of the chain. Figure 1 shows the price dynamics of our model when the two sellers are at distance $d_{\alpha \beta }=10$ on a chain of N=25 nodes. In Fig. 1a, we compare the results obtained under the two considered price update rules, namely the OS and the BR. Notice that under the OS price update rule, the dynamics converges to a local maximum of the two seller payoffs, which corresponds to the Hotelling fixed price solutions in Eqs. 2 and 3. We have verified that fixed prices are recovered for all the positions of the two sellers, except when they are too close and both on the same side of the chain (see Supplementary material). In the case where sellers update their prices according to the BR rule, the price dynamics is more complex than for the OS rule, and the model is able to generate Edgeworth’s price cycles (5, 45). In this case, the prices form closed stationary cycles with a peculiar asymmetric pattern. To highlight the features of such cycles in Fig. 1b, we report the typical trajectories of the two sellers’ prices as a function of time. We notice that the two sellers start undercutting the prices until a lower price bound when one of the two sellers suddenly increases the price. Such a seller is immediately followed by the other one, and then the price undercutting process starts again. For each cycle *i*, we have computed, as shown in Fig. 1c, the period $T_{i}$, defined as the number of time steps between two consecutive maxima, and the cycle amplitude $\delta_{i}$, given by the price difference between maximum and minimum. In Fig. 2, we study the BR price dynamics as a function of the positions of the two sellers on the chain. In particular, we report the average cycle period $\bar{T}$ (Fig. 2a,b) and its variance $\sigma_{T}$ (Fig. 2c,d), and the average amplitude $\bar{\delta }$ (Fig. 2e,f), as functions of the normalized distances $d_{\alpha \beta }^{\prime }=d_{\alpha \beta }/D$ and $d_{\alpha \beta }^{\prime \prime }=d_{\alpha \beta }^{\prime }C_{n_{\alpha }}/C_{n_{\beta }}$. Here, *D* is the chain diameter, that is the largest possible distance between two nodes in the chain, while $C_{n_{\alpha }}$ and $C_{n_{\beta }}$ are the closeness centralities (CCs) (29) of the two sellers, i.e. the inverse of the average distance between the position of a seller and all the nodes in the network. With such normalizations, both quantities $d_{\alpha \beta }^{\prime }$, $d_{\alpha \beta }^{\prime \prime }$ take value in the range [0,1]. Each data point in Fig. 2 represents a unique pair of seller positions $\left(n_{\alpha },n_{\beta }\right)$, with $n_{\alpha }<n_{\beta }$. Note that different pairs $\left(n_{\alpha },n_{\beta }\right)$ yield identical values of $d_{\alpha \beta }^{\prime }$ when the node pairs distance is the same, and identical values of $d_{\alpha \beta }^{\prime \prime }$ when the node pairs share also the same closeness centrality. An independent simulation run was conducted for each unique pair. According to the sellers’ positions, the model gives rise to four different types of stationary solutions, whose dynamics is illustrated in Fig. 3. For values of $d^{\prime \prime }<0.5$, we observe Edgeworth’s cycles (Fig. 3a) with average cycle period $\bar{T}$ and average amplitude $\bar{\delta }$ (Fig. 2b and f) that increase with $d^{\prime \prime }$ until $d^{\prime \prime }\approx 0.25$ (Fig. 3b), and then decrease until $d^{\prime \prime }\approx 0.5$ (Fig. 3c) . For $d^{\prime \prime }>0.5$, we have the coexistence of Hotelling fixed points (Fig. 3f) and irregularly oscillating solutions (with fluctuations of the order of the price unit) around Hotelling fixed points (Fig. 3e). The appearance of irregular oscillations at $d^{\prime \prime }\approx 0.5$ is revealed by the sharp change in the variance of the periods $\sigma_{T}$ in Fig. 2c and d. Finally, for $d^{\prime \prime }\approx 0.5^{-}$, together with the two solutions found for $d^{\prime \prime }>0.5$, we also observe the emergence of price cycles with broad amplitude as in Edgeworth cycles but irregular period as for the solutions oscillating around the Hotelling equilibrium (Fig. 3d). In such cycles, the two sellers follow a pattern that is inverted to that of the standard Edgeworth cycles. They spend most of the time at the Hotelling equilibrium, until one of the two sellers dramatically cuts its price to steal the buyers from the other seller, still making the same profit as before. The other seller sees its earnings dropping to zero and is then forced to follow the other seller in cutting the price to reacquire part of the market. Then, the two sellers start raising the prices, until they reach again the Hotelling equilibrium. Notice that *reverse Edgeworth cycles*, such as those theoretically predicted by our model, can indeed exist in real systems, as recently found in empirical analyses of online bid platforms (9).

### Bounded rationality

**Fig. 4.**
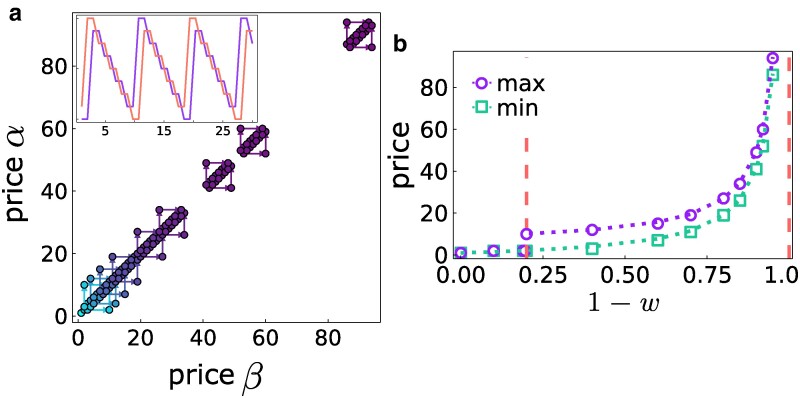
Price patterns for the BR dynamics and buyers’ bounded rationality, when both sellers are located on the same node. a) Edgeworth cycles corresponding, from left to right, to increasing values of bounded rationality 1−w. Since the two sellers are indistinguishable and positioned at the same location, their price cycles are identical. b) Numerical results (colored dots) align perfectly with the analytical predictions. In particular, the dashed vertical lines mark the domain in 1−w given by Eq. 4 where cycles are observed, while the green and purple dotted lines represent the minimum and maximum prices, respectively, as determined by Eqs. 5 and 6.

Let us now consider the case w<1, where a fraction of the buyers has no information on seller positions and prices. We present here the results for the symmetric case $n_{\alpha }=n_{\beta }$ (the two sellers *α* and *β* are on the same node), which allows a complete analytical characterization of the price dynamics under a BR price update (Fig. 4). We recall that the fraction *w* of informed buyers will buy from the cheaper seller while 1−w will buy with equal probability from the two sellers on the same node, as long as the price difference Δp is below a given threshold $\Delta p_{T}>0$, a free parameter representing the limit of bounded rationality. In fact, if the price difference exceeds $\Delta p_{T}>0$, buyers act rationally and choose to purchase from the lower-priced seller. Edgeworth cycles also emerge for w<1, depending on the values of *w* and of the limit of bounded rationality $\Delta p_{T}$. In particular, we observe a critical value of $w_{c}=1-2/(2+\Delta p_{T})$, such that Edgeworth cycles exist only for $w<w_{c}$ or otherwise if:


(4) $$\frac{2}{2+\Delta p_{T}}\leq 1-w\leq 1,$$

while the minimum and maximum prices in each cycle are given by (see Supplementary material for details):


(5) $$p_{m}=\frac{(1-w)\Delta p_{T}+1}{2w}+\frac{1}{2}$$

(6) $$p_{M}=p_{m}+\Delta p_{T}$$

It is worth stressing that these results hold for any network topology, as by placing the sellers on the same nodes we are neglecting the effect of the network structure, focusing only on the effect of bounded rationality.

This is not the case for the most general case w<1 and $n_{\alpha }\neq n_{\beta }$, that is where the two sellers occupy different nodes, where in this case, depending on the nodes occupied, the network structure can play a major role. However in this case, even in the simple scenario of a chain, the results are highly nontrivial and the emerging price dynamics are complex and of difficult theoretical interpretation. In particular, for the BR update rule, when the fraction of buyers with no information is small (1−w<0.1), we numerically observed that, as 1−w increases, the interval of values of $d^{\prime }$, the normalized distance between sellers, where we observe the Edgeworth cycles (as a reference, see Fig. 2 for the case 1−w=0) moves towards larger values of $d^{\prime }$ (i.e. to the right of the *x*-axis), with the Edgeworth cycles disappearing for smaller values of $d^{\prime }$. Further increasing 1−w (1−w≥0.1) still moves the interval of distance towards higher values of $d^{\prime }$, but at the same time, Edgeworth cycles appear again for small values of $d^{\prime }$ (left of the *x*-axis), with a sort of “Pac-Man” effect. In the Supplementary material, we provide a complete numerical characterization of the price dynamics for buyers with bounded rationality over a chain, both for the BR and OS price update rules.

### Real-world markets

**Fig. 5.**
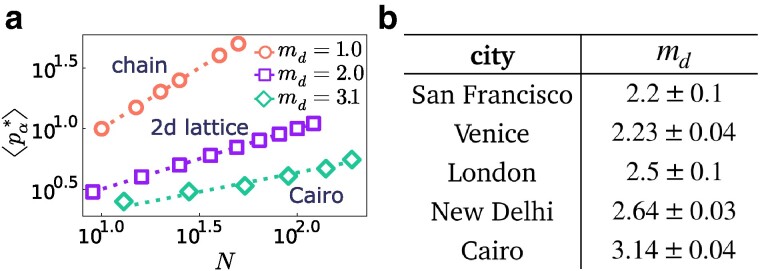
a) Maximum values $\left\langle p_{\alpha }^{*}\right\rangle$ of the average equilibrium price as a function of the graph order *N* for different networks: a chain, a regular square lattice and the real street network of a city (in this plot Cairo, Egypt). We report the corresponding market competition dimension $m_{d}$ obtained by fitting $\langle p_{\alpha }^{*}\rangle (N)=N^{1/m_{d}}$ to the data points. b) $m_{d}$ values for several cities. The reported error is the SE of the estimate.

In order to explore the effects that the topology of the market has on the price dynamics, we have studied our model on real-world markets. As examples of real-world markets, we have considered the spatial structures of urban street networks of 20 cities from all over the world (17, 18). Cities are characterized by different structural properties and layouts, and we have therefore investigated, through numerical simulations of our model, how their topology impacts the dynamics of prices. We focus on the case of a perfectly rational population of buyers, namely w=1, and we look at the average equilibrium price, $\langle p_{\alpha }^{*}\rangle_{d_{\alpha \beta }}$, where $\langle \cdot \rangle_{d_{\alpha \beta }}$ indicates the average over all the positions of the two sellers for a fixed distance $d_{\alpha \beta }$. Our numerical simulations show that, for sufficiently large values of $d_{\alpha \beta }$, the average equilibrium price reaches a maximum value $\left\langle p_{\alpha }^{*}\right\rangle$ (see Supplementary material). This value depends on the graph size *N* and the network topology but is independent of the type of price dynamics (namely OS or BR). For the results presented, we used the BR price dynamics. Figure 5a shows the scaling of $\left\langle p_{\alpha }^{*}\right\rangle$ with *N* for different networks. In particular, we compare a linear market (chain) to a 2D square lattice and to the street network of Cairo. For artificial networks (chains and square lattices), we can study this scaling simply by generating systems for increasing values of *N*. Instead, for real-world street networks, we examined subnetworks within neighborhoods of range *r* around the node with the highest closeness centrality, that is we included only nodes within fixed distance *r*. By incrementing *r*, we sampled subnetworks of increasing size *N* (see Supplementary material). By fitting the numerical results by $\langle p_{\alpha }^{*}\rangle (N)∼N^{1/m_{d}}$, it is possible to define the *competition dimension* of a market from the value of $m_{d}$ (see Materials and methods). In fact, the exponent $m_{d}$ quantifies how the maximum price (and payoff) increases with the order of the graph *N*. The higher $m_{d}$, the slower the maximum price increases, i.e. $m_{d}$ is a measure of the price competition. In particular, we obtained a value of $m_{d}=1$ for the linear market and $m_{d}=2$ for a square lattice. Hence, it is intuitive to identify $m_{d}$ as the dimension of a market. Figure 5b reports the values of $m_{d}$ obtained for the street networks of different cities (see Supplementary material for more details and for other cities). Interestingly, for all real-world street networks we found $2<m_{d}<3.2$, i.e. the price competition is enhanced with respect to a 2D square lattice, and the maximum prices increase considerably slower.

### Hotelling centrality

**Fig. 6.**
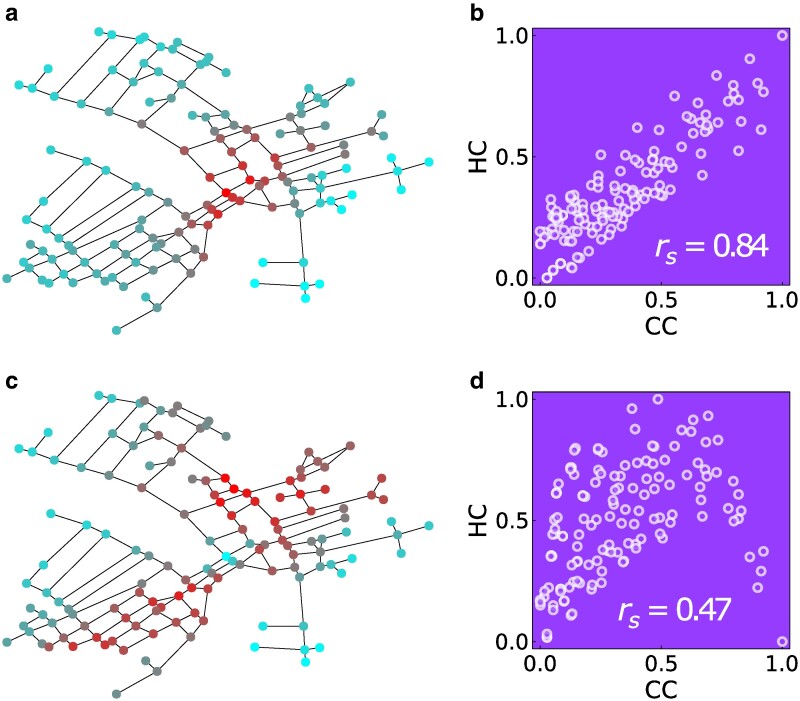
HC for the street network of Venice and its comparison with the CC. Both centralities are normalized between 0 and 1. a, b) refer to the OS dynamics, while c, d) to the BR dynamics. The Spearman’s correlation coefficient $r_{s}$ between the two quantities in the scatter plots is also reported.

The dynamics of our competition model can also be used to introduce a new measure of graph centrality (29). We define the so-called HC of a node, as the average payoff earned by a seller placed on that node, where the averages are taken over all the possible positions of the other seller. Hence, nodes with the highest HC are the best positions that a seller can occupy to maximize its earnings, when the seller has no information on the position of the other seller. Interestingly, in the case of a chain, we observe that the two nodes with the highest HC under BR dynamics are i=0.25N and j=0.75N (see Supplementary material). These positions, when occupied by the two sellers, minimize the average delivered cost for the buyers. Namely, the positions guaranteeing the average highest payoff to a seller are also the ones minimizing the transportation cost of the buyers. Figure 6 illustrates how the HC works in a real-world market, such as the street network of Venice (the area around Rialto bridge) (17, 18), and compares the HC with a standard measure of centrality, namely the closeness centrality (CC) defined as the inverse of the average distance between a given node and all the other nodes in the network. Figure 6a and c shows the spatial distribution of HC on the map of the city, with the nodes with the highest values of centrality colored in red. The scatter plots (HC, CC) in Fig. 6b and d indicates that, while HC is strongly correlated to CC in the case of the OS dynamics, the two measures can give different results when instead a BR dynamics is adopted. In the latter case, we observe that the relation between the HC of a node and its position on the graph is highly nontrivial. e.g. the red nodes with the highest HC in Fig. 6c are different from those with the highest CC (i.e. the nodes minimizing the average distance from the other nodes), which are located in a single area around the Rialto bridge (the graph barycenter (46)). They define instead two different centers (the two red areas) in the graph, one on each side of the bridge, while the bridge has low HC (colored in cyan).

## Discussion

In the dynamical model of price formation, we have introduced in the article, two sellers and a large number of buyers are placed over the nodes of a network. The buyers are modeled as random walkers with variable levels of information while the sellers are rational decision makers competing, through prices, for the market (i.e. for the buyers). In this way, the model accounts for the mutual influence between sellers’ price dynamics and buyers’ exploration dynamics of the market. Interestingly, depending on the market topology, i.e. on the structure of the underlying market, on the position of the two sellers’ and on the level of information available to the buyers, we are able to observe, within a single model, both fixed prices à la Hotelling and price cycles à la Edgeworth. In particular, we observe that, when the sellers are closer than a certain distance on the network, or when the fraction of rational buyers is below a critical threshold $w_{c}$, the fixed prices solutions are replaced by cycling patterns of prices. In addition to this, our numerical simulations show that, right at the transition between the region of fixed price solutions and that of regular Edgeworth price cycles, the model displays a completely novel type of cycles with a peculiar reversed pattern and irregular periods. These are similar to price patterns recently observed in online bid platforms (9). Notice that, in the classical Edgeworth model the market is a point, therefore price cycles do not depend on the market structure but instead emerge as a consequence of the sellers’ capacity constraints. That is, if one or both sellers alone cannot, or are not willing to, serve the entire market, there will be a spillover of buyers from the lower-priced seller to the higher-priced one. This means that the higher-priced seller, despite selling the same good for a less convenient price, will still obtain a positive profit. The presence of such a spillover destabilizes the market, causing a cyclic price war of attrition between the two sellers (5). Conversely, in our model the buyers spillovers towards the higher-priced seller emerge as a consequence of the market structure, the distance between the two sellers, and/or the bounded rationality of buyers. In this way, a structured market, or a localized market of buyers with bounded rationality, can give rise to cycling prices, even in the absence of sellers’ capacity constraints.

In addition to the theoretical results of our work, the versatility of representing markets as complex networks makes it possible to use our model to study the impact of the complex topology of real-world markets on price dynamics. In particular, we ran numerical simulations of our model on spatial street networks of cities from all over the world, which present a great variety of structural properties and layouts. Some of the cities we have considered, such as Barcelona, have patterns realized over a short period of time as the result of a single plan, and usually display a regular grid-like structure. Other cities, such as Ahmedabad, Cairo, and Venice, exhibit more complex patterns grown throughout a largely self-organized, fine-grained historical process, without any central control. Given this great variety, it is highly nontrivial to understand how the different structural properties of these spatial markets impact competition. Numerical simulations of our model show that competition on real-world spatial networks is always enhanced with respect to artificial 2D regular networks of the same size, with self-organized cities such as Ahmedabad, Cairo, and New Delhi, having particularly high values of competition dimension $m_{d}$.

In conclusion, understanding how the actual topology of a market impacts the price dynamics is of fundamental importance to design effective policies and to optimize the market both for sellers and consumers. Our dynamical model of price competition on networks, on the one hand reveals that the structure of real-world markets further amplifies the competition in price dynamics, on the other hand uses such dynamics to characterize the market topology. The model can also be easily generalized in different directions, e.g. considering more than two sellers, allowing both positions and prices to change at the same time, and including nonlinear costs of transportation (12, 47, 48). Since price competition is a fundamental mechanism of real-world markets, we hope our work can open new research avenues with tangible practical implications.

## Materials and methods

### Transition matrix and stationary distribution of buyers

The distribution of buyers over the *N* nodes of a graph, described by the row vector $\phi (t)=\{\phi_{i}(t){\}}_{i=1,\ldots ,N}$, evolves in time according to ϕ(t)=ϕ(t−1)Π, where the transition matrix $\Pi =\{\Pi_{ij}\}$ reads:


(7) $$\begin{array}{rl} \Pi_{ij} & =w\left[a_{ij}\frac{\sum_{\alpha }\sigma_{i\alpha }(j)\Theta (\min_{\beta }(P_{i\beta })-P_{i\alpha })}{\sum_{\alpha }\sigma_{i\alpha }\Theta (\min_{\beta }(P_{i\beta })-P_{i\alpha })}\right]\\ & \;+(1-w)\left(\frac{a_{ij}}{2}\frac{1}{k_{i}}+\frac{\delta_{ij}}{2}\right). \end{array}$$

Here, $a_{ij}$ are the entries of the adjacency matrix of the graph, where $a_{ij}=1$ if node *i* and *j* are connected by an edge (i.e. if node *j* can be reached from node *i*) and $a_{ij}=0$ otherwise. The quantities $\sigma_{i\alpha }$ and $\sigma_{i\alpha }(j)$ are, respectively, the total number of shortest paths from node *i* to seller *α*, and the number of those passing through node *j*. A shortest path between two nodes is any minimal sequence of edges connecting them (29). The Heaviside step function Θ(x) is such that Θ(x)=1 for x≥0, and zero otherwise. Therefore, in Eq. 7, Θ(x) is equal to 1 only if the delivered price $P_{i\alpha }$ of the seller *α* being considered in the sum is the lowest one, which means if it equals $\min_{\beta }(P_{i\beta }$), the minimum delivered price over the set of the sellers. Otherwise, it is zero and seller *α* does not contribute to the sum. Finally, $\delta_{ij}$ is the Kronecker delta, defined as $\delta_{ii}=1$ and $\delta_{ij}=0$ for j≠i. Equation 7 consists of two terms, corresponding to the two mechanisms contributing to the buyers’ random walk dynamics. The first term, weighted by the fraction *w* of rational buyers, describes the probability of a buyer moving from *i* to *j* following the shortest paths towards the seller offering the lowest delivered price. The second term, weighted by 1−w, accounts for the erratic movement of an uninformed or irrational buyer, which with probability 1/2 remains at the node *i*, and with probability 1/2 moves randomly from *i* to one of its $k_{i}$ neighbors. Since the Markov chain defined by this transition matrix is aperiodic, and the underlying graph is connected, a stationary distribution $\phi^{*}=\phi^{*}\Pi$ always exists unique (49) and can be easily found using a standard power method (50).

### Data sets of real-world spatial markets

As examples of real-world markets, we have considered the spatial structures of urban street networks. We used the data set provided in Refs. (17, 18), consisting of 1-square mile samples of the spatial street networks of 20 cities from all over the world. Each urban street network is represented as an undirected graph, where intersections are nodes and streets are edges. The number of nodes *N* and edges *K* goes from a minimum of N=169 and K=197 for the city of Walnut Creek to a maximum of N=2,870 and K=4,387 for the city of Ahmedabad. Cities differ in their structural properties and layouts (17, 18).

### Market competition dimension

The competition dimension $m_{d}$ of a market is the exponent characterizing how the maximum average seller’s price $\left\langle p^{*}\right\rangle$, which is equal to two times the maximum average seller’s payoff, scales with the size of the network *N*:


(8) $$\langle p^{*}\rangle =2\langle \pi^{*}\rangle ∼N^{1/m_{d}}.$$

In the case of artificial markets, we have directly generated chains and square lattices with different values of *N*. Instead, for urban street patterns, in order to produce networks with different *N* for each city, we have considered the subgraphs defined by a neighborhood of range *r* of the node with the highest closeness centrality, and we have varied the value of *r*. To calculate $m_{d}$, given a graph of size *N* and fixed positions of the two sellers, we averaged the payoff over the last 200 time steps of the model price dynamics, after waiting a thermalization time of 100 time steps. We then averaged the payoff over all the possible combinations of sellers’ positions at a given distance $d_{\alpha \beta }$, and we took the maximum over $d_{\alpha \beta }$ to obtain $\left\langle \pi^{*}\right\rangle$. We finally fitted the value of $m_{d}$ from the scaling relation in Eq. 8. See Supplementary material for more details and a complete list of the values of $m_{d}$ extracted for the 20 cities.

## Supplementary Material

pgaf014_Supplementary_Data

## Data Availability

The urban street networks dataset employed in this article is sourced from Refs. (17, 18) and is publicly available for download at https://www.complex-networks.net/datasets.html (29).

## Footnotes

a While, in principle, the BR should consider all (infinitely many) discrete prices, we verified numerically that in our model, when sellers are on different nodes and for *w* = 1, BR prices do not exceed twice the diameter of the graph. For*w <* 1, this limit can be exceeded depending on the value of *w*. This observation allows us to restrict our search for the BR price to this range of price values.

## Preprints

A preprint of this article is published at https://doi.org/10.48550/arXiv.2211.07412.

## References

[1] Moorthy KS . 1985. Using game theory to model competition. J Mark Res. 22(3):262–282.

[2] Vives X . Oligopoly pricing: old ideas and new tools MIT Press Books, The MIT Press, 2001.

[3] Cournot AA . Recherches sur les principes mathematiques de la theorie des richesses Hachette, Paris, 1838. English edition: *Researches into the mathematical principles of the theory of wealth*, translated by Bacon NT. MacMillan, New York, 1897.

[4] Bertrand J . 1883. Revue de la théorie de la recherche sociale et des recherches sur les principes mathématiques de la théorie des richesses. J Savants. 67:499–508.

[5] Edgeworth FY . The pure theory of monopoly. In: Papers relating to political economy. vol. 1. MacMillan, London, 1925. p. 111–142.

[6] Hotelling H . 1929. Stability in competition. Econ J. 39(153):41–57.

[7] Dudey M . 1992. Dynamic edgeworth-bertrand competition. Q J Econ. 107(4):1461–1477.

[8] Noel M . 2008. Edgeworth price cycles and focal prices: computational dynamic Markov equilibria. J Econ Manag Strategy. 17(2):345–377.

[9] Zhang XM, Feng J. 2011. Cyclical bid adjustments in search-engine advertising. Manage Sci. 57(9):1703–1719.

[10] Vickrey WS . Microstatics Harcourt, Brace & World, New York, 1964.

[11] Vickrey WS, Anderson SP, Braid RM. 1999. Spatial competition, monopolistic competition, and optimum product diversity. Int J Ind Organ. 17(7):953–963.

[12] d’Aspremont C, Gabszewicz JJ, Thisse J-F. 1979. On hotelling’s “stability in competition”. Econometrica. 47(5):1145–1150.

[13] Gal-or E . 1982. Hotelling’s spatial competition as a model of sales. Econ Lett. 9(1):1–6.

[14] Dasgupta P, Maskin E. 1986. The existence of equilibrium in discontinuous economic games, I: Theory. Rev Econ Stud. 53(1):1–26.

[15] Osborne MJ, Pitchik C. 1987. Equilibrium in Hotelling’s model of spatial competition. Econometrica. 55(4):911–922.

[16] Biscaia R, Mota I. 2013. Models of spatial competition: a critical review. Pap Reg Sci. 92(4):851–872.

[17] Crucitti P, Latora V, Porta S. 2006. Centrality measures in spatial networks of urban streets. Phys Rev E. 73(3):036125. 10.1103/PhysRevE.73.036125 16605616

[18] Cardillo A, Scellato S, Latora V, Porta S. 2006. Structural properties of planar graphs of urban street patterns. Phys Rev E. 73(6):066107. 10.1103/PhysRevE.73.066107 16906914

[19] Thurner S, Poledna S. 2013. DebtRank-transparency: controlling systemic risk in financial networks. Sci Rep. 3(1):1888. 23712454 10.1038/srep01888 PMC3664900

[20] Pichler A, et al 2023. Building an alliance to map global supply networks. Science. 382(6668):270–272. 37856603 10.1126/science.adi7521

[21] Simon HA . 1955. A behavioral model of rational choice. Q J Econ. 69(1):99–118.

[22] Stigler GJ . 1961. The economics of information. J Polit Econ. 69(3):213–225.

[23] Biely C, Thurner S. 2006. Statistical mechanics of scale-free networks at a critical point: complexity without irreversibility? Phys Rev E. 74(6):066116. 10.1103/PhysRevE.74.066116 17280130

[24] Funk S, Gilad E, Watkins C, Jansen VAA. 2009. The spread of awareness and its impact on epidemic outbreaks. Proc Natl Acad Sci U S A. 106(16):6872–6877. 19332788 10.1073/pnas.0810762106 PMC2672559

[25] Parkes DC, Wellman MP. 2015. Economic reasoning and artificial intelligence. Science. 349(6245):267–272. 26185245 10.1126/science.aaa8403

[26] Bruch E, Atwell J. 2015. Agent-based models in empirical social research. Sociol Methods Res. 44(2):186–221. 25983351 10.1177/0049124113506405 PMC4430112

[27] Zhan X-X, et al 2018. Coupling dynamics of epidemic spreading and information diffusion on complex networks. Appl Math Comput. 332:437–448. 32287501 10.1016/j.amc.2018.03.050 PMC7112333

[28] Newman MEJ . Networks Oxford University Press, 2018.

[29] Latora V, Nicosia V, Russo G. Complex networks: principles, methods and applications Cambridge University Press, 2017.

[30] Easley D, Kleinberg J. Networks, crowds, and markets: reasoning about a highly connected world Cambridge University Press, 2010.

[31] Thurner S, Klimek P, Hanel R. Introduction to the theory of complex systems Oxford University Press, 2018.

[32] Kirman A . 1997. The economy as an evolving network. J Evol Econ. 7(4):339–353.

[33] Rauch JE . 1999. Networks versus markets in international trade. J Int Econ. 48(1):7–35.

[34] Weisbuch G, Kirman A, Herreiner D. 2000. Market organisation and trading relationships. Econ J. 110(463):411–436.

[35] Rauch JE, Casella A, editors. Networks and markets Russell Sage Foundation, 2001.

[36] Kranton RE, Minehart DF. 2001. A theory of buyer-seller networks. Am Econ Rev. 91(3):485–508.

[37] Jackson MO . Social and economic networks Princeton University Press, 2008.

[38] Bramoullé Y, Kranton R, D’Amours M. 2014. Strategic interaction and networks. Am Econ Rev. 104(3):898–930.

[39] Coe NM, Wai-chung Yeung H. 2019. Global production networks: mapping recent conceptual developments. J Econ Geogr. 19(4):775–801.

[40] Scheffman DT, Spiller PT. 1987. Geographic market definition under the U. S. Department of Justice merger guidelines. J Law Econ. 30(1):123–147.

[41] Ulrick SW, Sacher SB, Zimmerman PR, Yun JM. 2020. Defining geographic markets with willingness-to-travel circles. Supreme Court Econ Rev. 28:241–284.

[42] Axelrod R . 1997. The dissemination of culture: a model with local convergence and global polarization. J Conflict Resolut. 41(2):203–226.

[43] Battiston F, Nicosia V, Latora V, Miguel MS. 2017. Layered social influence promotes multiculturality in the Axelrod model. Sci Rep. 7(1):1809. 28500281 10.1038/s41598-017-02040-4 PMC5431822

[44] Osborne MJ, Rubinstein A. A course in game theory The MIT Press, Cambridge, USA, 1994.

[45] Maskin E, Tirole J. 1988. A theory of dynamic oligopoly, II: Price competition, kinked demand curves, and edgeworth cycles. Econometrica. 56(3):571–599.

[46] West DB . Introduction to graph theory. 2nd ed Prentice Hall, Upper Saddle River, New Jersey, 2001.

[47] Economides N . 1993. Hotelling’s “main street” with more than two competitors. J Reg Sci. 33(3):303–319.

[48] Brenner S . 2005. Hotelling games with three, four, and more players. J Reg Sci. 45(4):851–864.

[49] Levin DA, Peres Y. Markov chains and mixing times. vol. 107. American Mathematical Society, 2017.

[50] Andrilli S, Hecker D. Chapter 9 - Numerical techniques. In: Elementary linear algebra, 4th ed. Academic Press, Burlington, MA, 2010. p. 608–615.

