# Supplementary material for: Dynamics of price formation on complex networks
Source: PNAS Nexus. 2025 Jan 31;4(1):pgaf014. doi: 10.1093/pnasnexus/pgaf014 (PMC11783569; doi:10.1093/pnasnexus/pgaf014)
Supplement: pgaf014_Supplementary_Data [file pgaf014_supplementary_data.pdf]

Supporting Information for

Dynamics of price formation on complex networks

Andrea Civilini and Vito Latora

Vito Latora.
E-mail: v.latora@qmul.ac.uk

This PDF file includes:

Figs. S1 to S7
Table S1
SI References

Discussion of the buyer dynamics

In our model we introduced a random-walk dynamics for buyers that combines two processes to represent buyer traffic in a market, allowing a tunable balance between attraction to points of interest (the sellers) and free navigation of the network. As mentioned in the main text, with probability $1-w$, buyers engage in a lazy random walk, while with probability w, they move towards the seller offering the lowest delivered price (i.e., defined as the sum of distance and price). The probability $1-w$ can be interpreted as either the likelihood of error in navigating towards the closest seller (for example due to limited information on seller locations and prices) or as a level of noise or "serendipity" in network navigation. When $w=1$, all buyers consistently visit the closest seller, as in the original Hotelling model, where buyers deterministically select the seller with the lowest delivered price. In the other extreme case, $w=0$, all buyers move through the network as random walkers, resulting in a stationary distribution proportional to the degree of the nodes (i.e., more congested nodes, crossroads, or websites are those with more incoming edges, roads, or links). In this scenario, the sellers' prices and positions have no influence over buyer dynamics and their stationary distribution. For intermediate values of w, the stationary distribution of buyers will be influenced by both mechanisms: while buyers will gravitate towards sellers, just a fraction of them will visit sellers, due to the level of randomness $1-w$. One can interpret intermediate values of w as describing crowd behaviour in a mall or on a city's main street, where shops attract people, even if not all visitors will end up shopping.

Analysis of the classical Hotelling solutions on a chain

Let us consider a chain of N nodes where the nodes are labelled with integer numbers in increasing order from 1 to N. We assume that the sellers α and β occupy respectively the positions (nodes) n_α, n_β such that $n_\alpha < n_\beta$ (being the sellers indistinguishable, the results can be readily extended to the symmetric case $n_\beta < n_\alpha$ by swapping the indices α and β). Moreover, because of the chain symmetry, we can focus in our analysis only on the positions n_α, n_β such that $n_\alpha < (N+1)/2$. All the results can be easily extended to the symmetric case $n_\alpha > (N+1)/2$. We start noticing that for fixed sellers' positions n_α, n_β, the sellers' payoffs as a function of prices are discontinuous in

$$p_\alpha = p_\beta - (n_\beta - n_\alpha) \quad [1]$$

$$p_\alpha = p_\beta + (n_\beta - n_\alpha) \quad [2]$$

In fact, when the absolute value of the price difference exceeds the distance between the two sellers, i.e. if

$$|p_\alpha - p_\beta| \geq n_\beta - n_\alpha \quad [3]$$

the payoff of the seller with the higher price suddenly drops to 0. Since the sellers are rational, they will never adopt prices such that $|p_\alpha - p_\beta| \geq n_\beta - n_\alpha$ during the price dynamics (or analogously such that $|p_\alpha - p_\beta| > n_\beta - n_\alpha - 1$, since the prices are discrete). That is, if we look at Fig. S1a the dynamics will be always confined between the two red lines representing $|p_\alpha - p_\beta| = n_\beta - n_\alpha$. This implies that if the classical Hotelling model's equilibrium

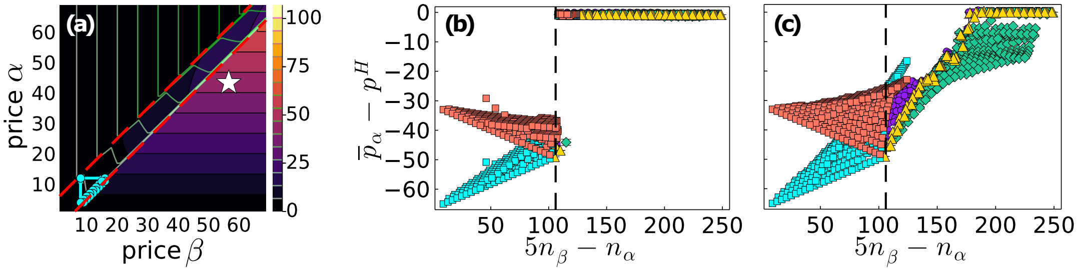

Fig. S1. Comparison of the analytical predictions with the simulations results for the price dynamics on a chain of $N = 50$ nodes. (a) The heatmap shows the payoff of seller α while the payoff's level curves of seller β are represented in green-magenta. The dashed red lines delimit the region where the price dynamics is confined to, i.e. where $|p_\alpha - p_\beta| \leq n_\beta - n_\alpha$. The white star represents the classical Hotelling equilibrium prices (p^H_α, p^H_β). As shown in this example for sellers positions $n_\alpha = 13$ and $n_\beta = 18$, when the classical Hotelling equilibrium prices, i.e. the white star, are outside the allowed domain for the price dynamics, the price trajectory (in cyan in the plot) cannot converge to them (in this case we show the trajectory under a Best Response price update rule). (b), (c) Difference between the equilibrium price averaged over time $\bar{p}_\alpha$ and the Nash equilibrium price predicted by the classical Hotelling model as a function of $5n_\beta - n_\alpha$, respectively for One-Step (OS) and Best Response (BR) dynamics. The dashed black lines represent the sufficient condition for not having convergence to the classical Hotelling prices, given by Eq. (8). The points are coloured according to the positions of sellers. In particular denoting the chain's middle point $x_c = 25.5$ and the sellers centre of mass $n_c = (n_\alpha + n_\beta)/2$, the orange and cyan squares are for positions (n_α, n_β) such that $n_\alpha < n_\beta < x_c$ and $n_\beta < n_\alpha < x_c$ respectively, while the purple circles, green diamonds and yellow triangles are for $n_\alpha < x_c < n_\beta$ with $n_c < x_c - 0.5$, $n_c > x_c + 0.5$ and $|n_c - x_c| \leq 0.5$ respectively.

$$p^H_\alpha = N + \frac{(a-b)}{3} \quad [4]$$

$$p^H_\beta = N - \frac{(a-b)}{3} \quad [5]$$

where $a = n_\alpha - 1$ and $b = N - n_\beta$, are such that

$$|p_\alpha^H - p_\beta^H| = \frac{2}{3}|a-b| = \frac{2}{3}|n_\alpha + n_\beta - N - 1| \geq n_\beta - n_\alpha \tag{6}$$

then the price dynamics cannot converge to them, because outside the region of prices involved in the price dynamics. We now investigate for which positions n_α, n_β this happens. Eq. (6) gives us two inequalities:

$$n_\beta \leq 5n_\alpha - 2(N+1), \textbf{ for } n_\alpha + n_\beta > N+1 \tag{7}$$

$$n_\alpha \geq 5n_\beta - 2(N+1), \textbf{ for } n_\alpha + n_\beta < N+1 \tag{8}$$

That is

$$N + 1 - n_\alpha < n_\beta \leq 5n_\alpha - 2(N+1) \tag{9}$$

$$5n_\beta - 2(N+1) \leq n_\alpha < N + 1 - n_\beta \tag{10}$$

However, looking at the first of these inequalities we notice that in order to have $N + 1 - n_\alpha < 5n_\alpha - 2(N+1)$ we need $n_\alpha > (N+1)/2$. Since in our analysis we are considering $n_\alpha < (N+1)/2$, we can focus exclusively on Eq. (10). By solving $5n_\beta - 2(N+1) < N + 1 - n_\beta$, we found the necessary condition that must be satisfied in order to have a non-empty set of n_α values satisfying Eq. (10):

$$n_\beta < \frac{N+1}{2} \tag{11}$$

That is, all the couples of positions (n_α, n_β) with $n_\alpha < n_\beta$ and $n_\alpha < (N+1)/2$ such that $\exists n_\alpha$ which satisfies Eq. (10) are among the positions where $n_\beta < \frac{N+1}{2}$, i.e. when both sellers are on the same side of the chain (that is, among the orange and cyan points in our plots). We can also found a sufficient condition for Eq. (10), by looking when $5n_\beta - 2(N+1) < 0$ (in this case all the values of n_α automatically satisfy Eq. (10), since $0 < n_\alpha$ by definition). In this way, we find the sufficient condition:

$$n_\beta < \frac{2}{5}(N+1) \tag{12}$$

If instead we are interested in the symmetric case where $n_\alpha > (N+1)/2$, to obtain the sufficient condition equivalent to Eq. (12) we just need to swap the index β with α, the $<$ sign with $>$ and subtract $N+1$ to the right-hand side of the inequalities, obtaining: $n_\alpha > \frac{3}{5}(N+1)$ (and where as usual $n_\alpha < n_\beta$). It is crucial to notice that Eq. (10) tells us the condition that must be satisfied for the Hotelling solution to be outside of the allowed domain of the price dynamics, but this is only a sufficient condition for not observing the classical Hotelling equilibrium. That is, it does not imply that if the Hotelling solutions are instead inside the domain interested by the price dynamics then the prices will converge to them. In particular, with the numerical simulations we found that for the One-Step dynamics the condition is actually necessary and sufficient for not observing the classical Hotelling solutions, i.e. all and only the positions for which the actual model price dynamics does not converge to the classical Hotelling solution satisfy Eq. (10), as shown in Fig. S1b. Instead, in Fig. S1c we see that for the Best Response dynamics the price dynamics does not converge to the classical Hotelling solutions even for positions which do not satisfy Eq. (10).

One-step price dynamics convergence to the classical Hotelling equilibrium

We consider the case where the sellers are allowed to change their price by ± 1 at each time step (i.e. when sellers use the One-Step, OS, price update rule) and we prove that the price dynamics converges to the classical Hotelling equilibrium (when it can, see previous SI section). Let us consider two sellers α and β respectively on node n_α and n_β of a chain of N nodes, where the nodes are labelled with integers numbers $1 \leq n \leq N$ in increasing order. We define the *indifference distance* d_{in} as the distance between seller α and the node with equal delivered price from the two sellers:

$$d_{in} = \frac{d_{\alpha\beta}}{2} + \frac{p_\beta - p_\alpha}{2} \tag{13}$$

where $d_{\alpha\beta} = n_\beta - n_\alpha$ is the distance between the two sellers and p_α, p_β are their prices. Fixed the positions of sellers (i.e. their distance), the buyers' flux ϕ (i.e. the share of buyers market) attracted by each seller at time t is a function of d_{in}^t the current indifference distance, i.e. of their current prices:

$$\phi_\alpha^t = a + d_{in}^t + \frac{1}{2} = n_\alpha + d_{in}^t - \frac{1}{2} \tag{14}$$

$$\phi_\beta^t = b + +d_{in}^t + \frac{1}{2} = N - n_\beta + d_{\alpha\beta} - d_{in}^t + \frac{1}{2} \tag{15}$$

where $a = n_\alpha - 1$ and $b = N - n_\beta$. It is worth pointing out that these equations for the buyers fluxes apply only for $|p_\beta - p_\alpha| < n_\beta - n_\alpha$ with $n_\alpha < n_\beta$. The payoff π of each seller can be readily found by multiplying the buyers market share by the respective prices at time t p_α^t and p_β^t:

$$\pi_\alpha^t = \left(n_\alpha + d_{in}^t - \frac{1}{2}\right) p_\alpha^t \tag{16}$$

$$\pi_\beta^t = \left(N - n_\beta + d_{\alpha\beta} - d_{in}^t + \frac{1}{2}\right) p_\beta^t \tag{17}$$

Let us focus on seller α and suppose that at time t it changes the price $p_\alpha^{t+1} = p_\alpha^t \pm 1$. As a consequence the indifference distance at time $t+1$ will be $d_{in}^{t+1} = d_{in}^t \mp 1/2$, since by increasing (decreasing) the price by 1, seller α decreases (increases) its buyers' market share by 1/2. In particular, by decreasing the price the payoff at time $t+1$ becomes

$$\pi_{\alpha,-}^{t+1} := \pi_\alpha^{t+1}(p_\alpha^t - 1) = \left(n_\alpha + d_{in}^{t+1} - \frac{1}{2}\right)\left(p_\alpha^t - 1\right) = \left(n_\alpha + d_{in}^t + \frac{1}{2} - \frac{1}{2}\right)\left(p_\alpha^t - 1\right) = \pi_\alpha^t + \frac{1}{2}p_\alpha^t - \left(n_\alpha + d_{in}^t\right) \quad [18]$$

while increasing the price by 1 the seller earns

$$\pi_{\alpha,+}^{t+1} := \pi_\alpha^{t+1}(p_\alpha^t + 1) = \left(n_\alpha + d_{in}^{t+1} - \frac{1}{2}\right)\left(p_\alpha^t + 1\right) = \left(n_\alpha + d_{in}^t - \frac{1}{2} - \frac{1}{2}\right)\left(p_\alpha^t + 1\right) = \pi_\alpha^t - \frac{1}{2}p_\alpha^t + \left(n_\alpha + d_{in}^t - 1\right) \quad [19]$$

We now compare Eq. (18), Eq. (19) to Eq. (16) to see when it is convenient for the seller α to keep the price fixed and when instead is more convenient to change it, i.e. when:

$$\pi_\alpha^t > \pi_{\alpha,-}^{t+1} = \pi_\alpha^t + \frac{1}{2}p_\alpha^t - \left(n_\alpha + d_{in}^t\right) \quad [20]$$

$$\pi_\alpha^t > \pi_{\alpha,+}^{t+1} = \pi_\alpha^t - \frac{1}{2}p_\alpha^t + \left(n_\alpha + d_{in}^t - 1\right) \quad [21]$$

That is

$$\frac{1}{2}p_\alpha^t - \left(n_\alpha + d_{in}^t\right) < 0 \quad [22]$$

$$-\frac{1}{2}p_\alpha^t + \left(n_\alpha + d_{in}^t - 1\right) < 0 \quad [23]$$

By substituting Eq. (13) we obtain

$$2p_\alpha^t - p_\beta^t < d_{\alpha\beta} + 2n_\alpha \quad [24]$$

$$2p_\alpha^t - p_\beta^t > d_{\alpha\beta} + 2(n_\alpha - 1) \quad [25]$$

These two inequalities combined together give us the condition for which it is not convenient for the seller α to change the price, i.e. when the price update dynamics stops:

$$d_{\alpha\beta} + 2(n_\alpha - 1) < 2p_\alpha^t - p_\beta^t < d_{\alpha\beta} + 2n_\alpha \quad [26]$$

And since the prices are discrete and the minimum price variation is $\Delta p \pm 1$, this implies:

$$2p_\alpha^t - p_\beta^t = d_{\alpha\beta} + 2n_\alpha - 1 \quad [27]$$

For seller β we can find an equivalent condition. In particular, by taking advantage of the symmetry of the chain it is sufficient to swap in Eq. (26) indices α and β and to replace $n_\alpha - 1$ with $N - n_\beta$:

$$d_{\alpha\beta} + 2(N - n_\beta) < 2p_\beta - p_\alpha < d_{\alpha\beta} + 2(N - n_\beta + 1) \quad [28]$$

That gives us:

$$2p_\beta^t - p_\alpha^t = d_{\alpha\beta} + 2(N - n_\beta) + 1 \quad [29]$$

From Eq. (27) we obtain $p_\beta = 2p_\alpha - d_{\alpha\beta} - 2n_\alpha + 1$, and by substituting it and $d_{\alpha\beta} = n_\beta - n_\alpha$ in Eq. (29) we finally arrive to the stationary price for seller α under OS price update:

$$p_\alpha^* = N + \frac{(n-1) - (N - n_\beta)}{3} = N + \frac{a-b}{3} \quad [30]$$

since by definition $a = n_\alpha - 1$ and $b = N - n_\beta$. Analogously we can obtain p_α from Eq. (29), and by replacing in Eq. (27) we find:

$$p_\beta^* = N - \frac{(n-1) - (N - n_\beta)}{3} = N - \frac{a-b}{3} \quad [31]$$

Therefore the stationary prices under the OS dynamics coincides with the classical Hotelling equilibrium prices, at least for sellers' positions such that the price dynamics can converge to the classical Hotelling solutions (i.e. when Eq. (10) holds).

Edgeworth cycles for bounded information and sellers on the same node

We assume that a fraction $1-w$ (where $0<w<1$) of the buyers has bounded information regarding the sellers' positions and prices. For this analytical treatment, we assume that the two sellers α and β are on the same node. We recall from the main text that the fraction w of informed buyers will buy from the cheaper seller while $1-w$ will buy with equal probability from the two sellers on the same node, as long as the price difference Δp is below the threshold $\Delta p_T > 0$. We can write the normalized (i.e. divided by the cardinality N of the network) fluxes of buyers attracted by the two sellers as a function of the price difference. If $p_\alpha = p_\beta$, each seller attracts half of the total market $\phi_\alpha = \phi_\beta = \frac{1}{2}$. If $0 < p_\beta - p_\alpha \leq \Delta p_T$,

$$\phi_\alpha = w + \frac{1-w}{2} = \frac{1+w}{2}, \quad [32]$$

$$\phi_\beta = \frac{1-w}{2}, \quad [33]$$

while if $p_\beta - p_\alpha > \Delta p_T$, α get all the buyers, i.e. $\phi_\alpha = 1$ and $\phi_\beta = 0$. The sellers' payoffs are readily found by multiplying the fluxes of buyers for the respective prices p_α and p_β. It is worth noticing that the case $p_\beta - p_\alpha > \Delta p_T$ clearly will never occur if sellers are rational, since by decreasing the price difference below the critical threshold seller β will earn some positive payoff instead of a null one. Hence we can focus on the case where the price difference is below Δp_T. In particular, we will compare the sellers' payoffs to understand when for a seller is convenient to keep the same price of the competitor and when instead it is convenient to raise its price above, or decrease it below, the competitor's price. We focus on seller α, considering the price of β fixed. Since the sellers are identical (they are both on the same node) the same analysis applies if we swap sellers' indices. We first notice that it is never convenient for the seller α to lower the price more than the minimum discrete price (i.e. a unit of price) below the competitor price, since its share of the market is equal to $\phi_\alpha = \frac{1+w}{2}$ for all prices below p_β, while instead the payoff earned $\pi_\alpha = \phi_\alpha p_\alpha$ increases linearly with p_α. That is, if a rational seller α decreases its price below p_β, it will adopt $p_\alpha = p_\beta - 1$. For the same reason, since increasing p_α above p_β guarantees a buyers market $\phi_\alpha = \frac{1-w}{2}$ for every price $p_\alpha > p_\beta$ (as long as $0 < p_\alpha - p_\beta \leq \Delta p_T$), if it is convenient for α to increase the price then the best payoff it can obtain is for the maximum price difference below the threshold, i.e. for $p_\alpha = p_\beta + \Delta p_T$.

- **Matching or beating opponent's price?**

  That is, we have to check when:

$$\frac{p_\beta}{2} > \left(\frac{1+w}{2}\right)(p_\beta - 1), \quad [34]$$

where on the LHS we have the payoff of seller α for adopting the same price of seller β, on the RHS the payoff for a price $p_\alpha = p_\beta - 1$. With some basic manipulation, we obtain:

$$1 - w > \frac{p_\beta - 2}{p_\beta - 1}. \quad [35]$$

Or analogously, focusing on the price as a function of w, we obtain:

$$p_\beta < \frac{1+w}{w}. \quad [36]$$

Since the fraction of uninformed buyers is strictly greater than zero,Eq. (35) implies that if $p_\beta = 2$ there is no value of $1-w>0$ for which is convenient to lower the price further (i.e. the minimum possible price is $p\_ = 2$).

- **Matching or exceeding opponent's price?**

  In this case, we have to verify when

$$\frac{p_\beta}{2} > \left(\frac{1-w}{2}\right)(p_\beta + \Delta p_T), \quad [37]$$

where on the LHS there is the payoff of seller α for adopting the same price of seller β, on the RHS the payoff for a price $p_\alpha = p_\beta + \Delta p_T$. This gives us:

$$1 - w < \frac{p_\beta}{p_\beta + \Delta p_T}. \quad [38]$$

Or if we focus on the price as a function of w:

$$p_\beta > \frac{(1-w)\Delta p_T}{w}. \quad [39]$$

- **Beating or exceeding opponent's price?**

  In this case, we have to verify when

$$\frac{1+w}{2}(p_\beta - 1) > \left(\frac{1-w}{2}\right)(p_\beta + \Delta p_T), \quad [40]$$

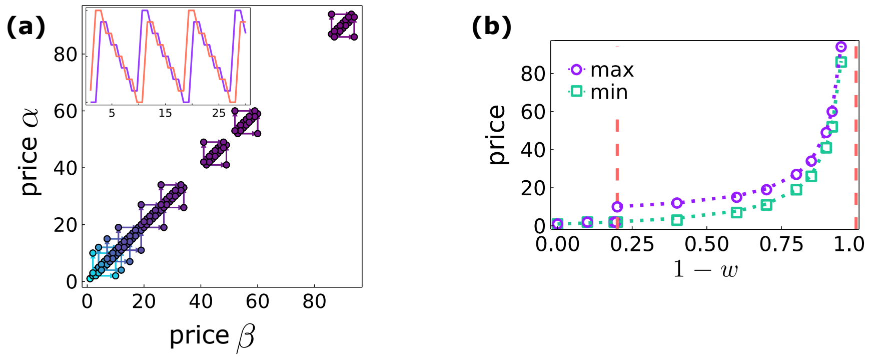

Fig. S2. (a), (b) Edgeworth cycles as a function of $0 < (1-w) < 1$, i.e. for bounded buyers' rationality, when both sellers are on the same node. In this case, the price cycles are identical for the two sellers (being the sellers indistinguishable and on the same node). (b) We observe that the numerical results (coloured dots) are in perfect agreement with the analytical predictions. In particular, the dashed vertical lines delimit the domain in $1-w$ given by Eq. (45) where cycles exist, while the green and purple dotted lines are respectively the minimum and maximum prices given by Eq. (46), Eq. (47).

where on the LHS we have the payoff of seller α for adopting a price $p_\alpha = p_\beta - 1$, on the RHS the payoff for a price $p_\alpha = p_\beta + \Delta p_T$. This gives us:

$$1 - w < \frac{2(p_\beta - 1)}{2p_\beta + \Delta p_T - 1}. \tag{41}$$

Or if we focus on the price:

$$p_\beta > \frac{(1-w)\Delta p_T + 1}{2w} + \frac{1}{2} =: p_{th}. \tag{42}$$

This means that as long as the price of the competitor is higher than this critical threshold p_{th} the sellers will undercut the competitor's price by one. Once this critical threshold is reached then one of the sellers (e.g. seller α) will increase its price to the maximum allowed, i.e. to $p_\alpha = p_{th} + \Delta p_T$. However, from Eq. (35) we know that the minimum price reachable undercutting the competitor is $p\_ = 2$, otherwise for all $1 - w > 0$ it is more convenient for the sellers to charge the same price than to reduce the price further. By substituting $p\_ = 2$ in Eq. (41) we found that if

$$(1-w) < \frac{2(p\_ - 1)}{2p\_ + \Delta p_T - 1} = \frac{2}{3 + \Delta p_T} =: (1-w)', \tag{43}$$

then $p_{th} < 2$. That is, for $(1-w) < (1-w)'$ the price undercutting process will stop when both sellers reach the same price $p\_ = 2$. At this point, we have to check the condition given by Eq. (37), to see if for a seller it is more convenient to stay at the same price of the competitor $p\_ = 2$ or to rise its price. Substituting $p\_ = 2$ in Eq. (38) we see that, as long as

$$1 - w \geq \frac{2}{2 + \Delta p_T} =: (1-w)'', \tag{44}$$

it is more convenient (or it is indifferent, in case of equal sign) to raise the price. Otherwise, since $(1-w)' < (1-w)''$, the price dynamics remains stuck in $p_\alpha = p_\beta = p\_ = 2$. Hence, for sellers on the same node, Edgeworth cycles exist for

$$\frac{2}{2 + \Delta p_T} \leq 1 - w \leq 1, \tag{45}$$

while the minimum and maximum prices in each cycle are given by:

$$p_m = \frac{(1-w)\Delta p_T + 1}{2w} + \frac{1}{2}, \tag{46}$$

$$p_M = \frac{(1-w)\Delta p_T + 1}{2w} + \frac{1}{2} + \Delta p_T. \tag{47}$$

In Fig. S2 we compare the theoretical predictions with the simulation results, finding a perfect agreement.

Bounded information and sellers on different chain's nodes

We characterize numerically the impact of w over the price dynamics, both for the BR (Figs. S3, S4) and OS (Fig. S5) price updates, as a function of the sellers positions on a chain. In particular, in Fig. S3 and Fig. S4 we report respectively the average amplitude of the price cycles $\overline{\delta}_\alpha$ and the average price $\overline{p}_\alpha$ as a function of the normalized distance between the two sellers $d' = d_{\alpha\beta}/D$, where D is the chain's diameter (for these results we used a chain of $N = 15$ nodes). Each panel refers to a different value of w, the fraction of informed buyers. It is worth noticing that the points with $\overline{\delta}_\alpha = 0$ correspond to fixed points of the price dynamics, while the points with $\overline{\delta}_\alpha > 0$ correspond to price cycles. As the fraction of informed buyers decreases (i.e., $1-w$ increases), we observe that the price cycles move towards higher values of d'. It is interesting to notice that even

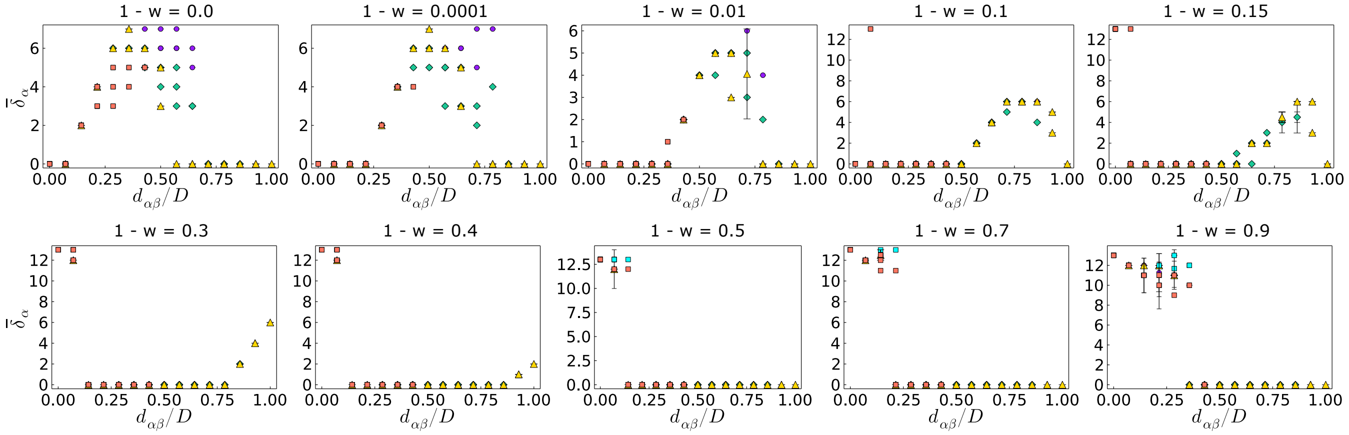

Fig. S3. Numerical results for BR price dynamics. In particular, the panels show the average amplitude of the price cycles $\overline{\delta}_\alpha$ as a function of the normalized distance between the sellers $d' = d_{\alpha\beta}/D$, where D is the chain's diameter (these results have been obtained for a chain of $N = 15$ nodes). Each panel refers to a different value of w, the fraction of informed buyers. The points with $\delta_\alpha = 0$ correspond to fixed points of the price dynamics.

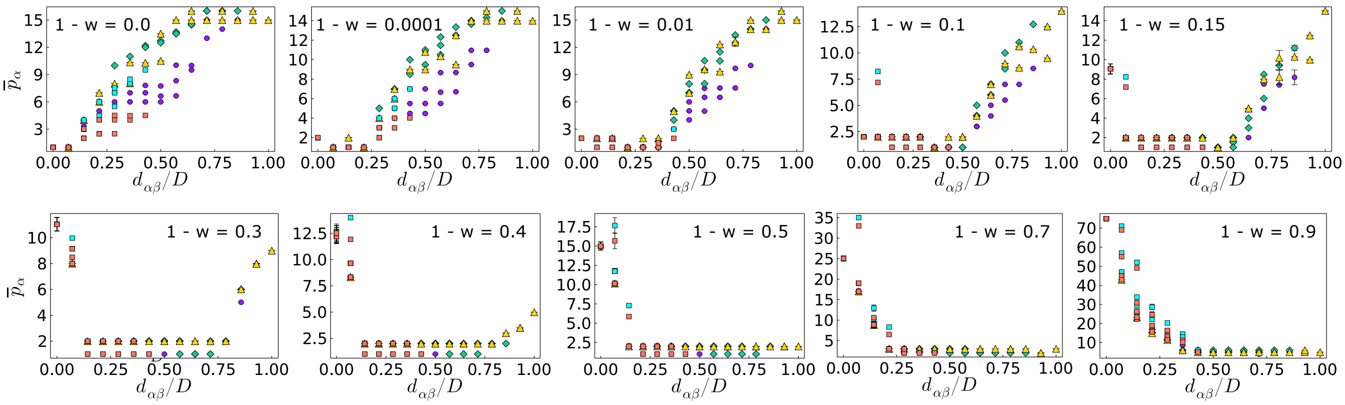

Fig. S4. Numerical results for BR price dynamics. In particular, we report the average price $\overline{p}_\alpha$ as a function of the normalized distance between the sellers $d' = d_{\alpha\beta}/D$, where D is the chain's diameter (these results have been obtained for a chain of $N = 15$ nodes). Each panel refers to a different value of w, the fraction of informed buyers.

very small values of $0 < 1 - w \leq 0.01$, i.e. when just a small fraction of the buyers is uninformed about sellers' prices and positions, have a noticeable impact on the price dynamics. For values of $1 - w$ larger than ≈ 0.1 we observe that price cycles appear for small values of d'. By further increasing $1 - w$, cycles gradually disappear for large values of d', while on the left of the d' axis cycles extend to larger d'. In Fig. S4 we can observe that for $w > 0.1$ the average prices of the cycles are typically higher than the prices for the fixed points. Fig. S5 shows the difference between the average prices for OS price dynamics and the classical Hotelling Nash equilibrium (1) prices given by Eq. (4), Eq. (5), as a function of $5n_\beta - n_\alpha$ (see first section of the SI). For $w = 1$ we observe that the sufficient condition given by Eq. (8) for not having convergence to the classical

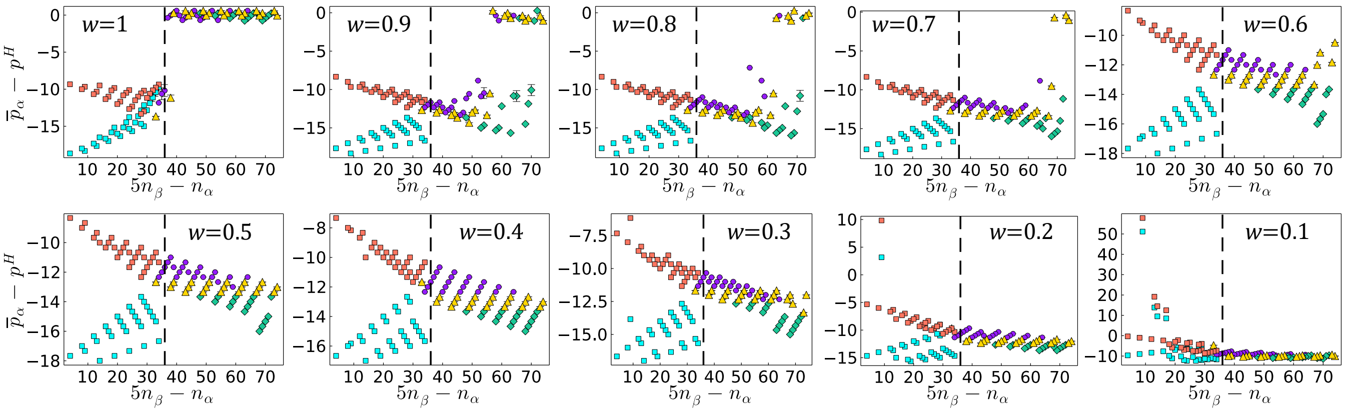

Fig. S5. Difference between the average prices for OS dynamics and the classical Hotelling Nash equilibrium prices given by Eq. (4), Eq. (5), as a function of $5n_\beta - n_\alpha$ (see first section of the SI). Each panel refers to a different value of w, the fraction of informed buyers. The vertical dashed lines mark the sufficient condition Eq. (8) for not having convergence to the classical Hotelling equilibrium prices.

Hotelling equilibrium prices is also a necessary condition, as shown in the first section of the SI. As w decreases we observe that more and more points (for increasing values of $5n_\beta - n_\alpha$) diverge from the classical Hotelling solutions. Interestingly, our numerical simulations show that, when this divergence initially occurs, the measured average price is always smaller than the one predicted by the classical Hotelling model. Further decreasing w (e.g. for $0.1 \leq w \leq 0.2$, as shown in Fig. S5) we observe that for small values of $5n_\beta - n_\alpha$ some points start showing increased payoff respect to the classical Hotelling prediction. It is worth noticing that the case $w = 0$, i.e. when we have just uninformed buyers, is trivial: the prices will increase indefinitely, independently from the sellers' positions, since the sellers can gradually increase their prices (keeping the prices difference below the threshold Δp_T, introduced in the main text) without any reaction from the buyers.

Extracting the market competition dimension

It is trivial to prove that on a linear market, given Eq. (4), Eq. (5) and $d_{\alpha\beta} = N - a - b$, the expected price $\langle p_\alpha^H \rangle_{d_{\alpha\beta}}$ for a distance $d_{\alpha\beta}$ is actually independent on $d_{\alpha\beta}$ and it is simply equal to $\langle p_\alpha^H \rangle_{d_{\alpha\beta}} = \langle p_\alpha^H \rangle = N$, where $\langle \cdot \rangle_{d_{\alpha\beta}}$ indicates the average over all the sellers positions at distance $d_{\alpha\beta}$. However, as we have shown in the first section of the SI, the classical Hotelling solutions are not expected to hold for all the positions of the sellers. In particular, on a chain market of order N we observe that the expected equilibrium price is a function of the sellers' distance and it converges to a maximum value $\langle p_\alpha^* \rangle \sim N$ for both OS and BR dynamics. For market structures more complex than a simple line (even a regular square lattice) finding

the equilibrium prices is not trivial, if possible at all, hence we rely on numerical simulations for the characterization of the equilibrium prices. In Fig. S6 we can observe that $\langle p^*_\alpha \rangle$ is indeed a function of N and of the market structure (i.e. the topology of the graph). To measure the market competition dimension m_d we first found the maximum of the average price $\langle p^*_\alpha \rangle$ as a

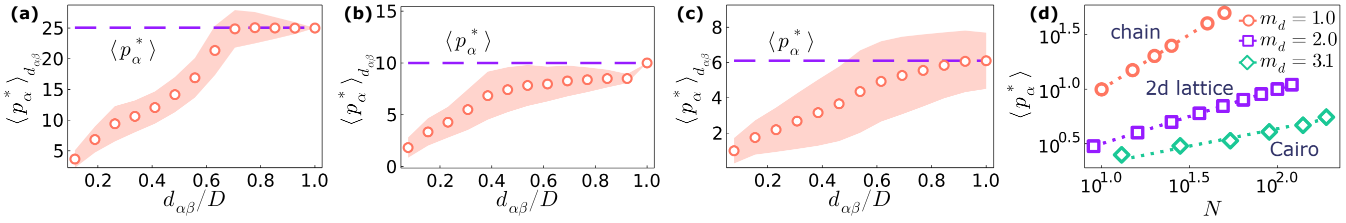

Fig. S6. Maximum average equilibrium price $\langle p^*_\alpha \rangle$ for different network topologies and its scaling with the network order N. For these plots, we used the BR price update rules. Panels (a), (b) and (c) refer respectively to a chain of $N = 25$ nodes, a square lattice of $N = 100$ nodes (i.e. a 10x10 grid) and the street networks of the city of Cairo with $N = 190$ nodes (it has been obtained taking a neighbourhood of range $r = 7$ of the node in the original dataset with the highest closeness centrality (2, 3) (for more details see next section of the SI). (d) shows the values of $\langle p^*_\alpha \rangle$ for the three different networks as a function of the network order N and reports the market competition dimensions m_d obtained by fitting the scaling relation.

function of the seller distance for different values of the network order N. In the case of artificial networks (i.e. for chains and square lattices) we obtained networks of different order N directly by generating chains and square lattices for different values of N. Instead for the street networks of real-world cities, we have considered the subgraphs defined by a neighbourhood of range r of the node with the highest closeness centrality for different values of r (for more details see the next section of the SI). Then we fitted the numerical results with the the scaling relation (see main text):

$$\langle p^* \rangle \sim N^{1/m_d} \qquad [48]$$

Market competition dimension for real-world street networks

We report the market competition dimension m_d for the street networks of real-world cities. For this measure, we used a dataset of the street networks of 19 cities from all over the world (4, 5). We recall from the main text that m_d is defined by

$$\langle p^* \rangle = 2\langle \pi^* \rangle \sim N^{1/m_d} \qquad [49]$$

and it represents how the maximum average seller's price (which is equal to two times the maximum average seller's payoff) as a function of the sellers' distance scales with N, the cardinality of the market's network. The averages are performed over all the possible combinations of sellers' positions at a given distance $d_{\alpha\beta}$. We performed this measure with two different methods to check the consistency of our results. We first sampled a subgraph of the street networks: starting from the more central node in the graph, measured as the node with the highest closeness centrality, we took the subgraph defined by a neighbourhood of range r, for $2 \le r \le 7$. Obviously, increasing r also N the number of nodes in the subgraph increases. The choice of a maximum value of $r = 7$ has two motivations. The first one is related to the dataset we used: since the street network dataset of each city represents a geographical map of finite size, in most of the cities for $r > 7$ we are already hitting the edges of the finite map. Therefore further increasing r (and hence $N(r)$) we would not observe how the market expands with N since the expansion is bounded by the finiteness of the map. Instead, for $2 \le r \le 7$ we have a consistent range of $N(r)$ spanning more than one order of magnitude (from $N(r) \approx 10$ to $N(r) \approx 100$) for all cities, without reaching the edges of the street maps. The second is instead a practical reason: since we have to run simulations of the price dynamics for all the possible combinations of positions of the two sellers, the possible combinations increase roughly as N^2 and so increasing further $N(r)$ would have brought to impractical simulations' length. For each subgraph of size $N(r)$ we measured $\langle p^* \rangle(N) = 2\langle \pi^* \rangle(N)$ as a function of the sellers' distance. Given a graph of size N, to find this maximum we first averaged the payoff over the last 200 time steps of the price dynamics after waiting a thermalization time of 100 time steps, for each couple of sellers' positions. Then we averaged over all the possible combinations of sellers' positions at a given distance $d_{\alpha\beta}$ and we took the maximum over $d_{\alpha\beta}$ of the resulting average payoff. It is worth noticing that for these results we used the BR price dynamics, however, the maximum average payoff (i.e. price) does not depend on the specific dynamics (i.e. BR or OS) but only on the specific network. Hence for each city, we fitted the values $\langle p^* \rangle$ obtained for the different N by $\langle p^* \rangle \sim N(r)^{1/m_d}$ to find the market competition dimension m_d. The second method that we used, which we will refer to as the *brute force* method, is to directly obtain m_d for the largest sample of size N_{max} (i.e. the one for $r = 7$) from $\langle p^* \rangle = N_{max}^{1/m_d}$, as $m_d = \log(N_{max})/\log(\langle p^* \rangle)$. We report in Table S1 the fitted values of m_d. The results show a very good agreement between the two methods for all cities, despite the brute force method being obviously less refined. We recall that m_d for a chain is $m_d^{chain} = 1$, while for a regular square lattice is equal 2. We notice that for all cities the fitted value of m_d is greater than 2. In particular, for the cities in our dataset, we found $2.1 \le m_d \le 3.14$.

Hotelling centrality and average distance from the closest seller on a chain

In this section, we show that on a linear market represented as a chain graph, the nodes which maximise the Hotelling centrality (HC) under BR dynamics are those which minimise $\overline{d}_n$, the average distance of the buyers from the closest seller given the

city	$N(r)^{1/m_d}$ fit	N_{max}^{1/m_d} fit
Ahmedabad	2.6 ± 0.1	2.5 ± 0.1
Barcelona	2.29 ± 0.03	2.23 ± 0.03
Bologna	2.41 ± 0.09	2.3 ± 0.1
Brasilia	2.44 ± 0.03	2.45 ± 0.04
Cairo	3.14 ± 0.04	3.06 ± 0.09
Irvine	2.4 ± 0.1	2.3 ± 0.1
London	2.48 ± 0.07	2.46 ± 0.05
Los Angeles	2.6 ± 0.1	2.4 ± 0.1
New Delhi	2.64 ± 0.03	2.55 ± 0.07
New York	2.28 ± 0.02	2.26 ± 0.04
Paris	2.5 ± 0.1	2.4 ± 0.1
Richmond	2.7 ± 0.1	2.6 ± 0.1
San Francisco	2.2 ± 0.1	2.1 ± 0.1
Savannah	2.53 ± 0.04	2.53 ± 0.04
Seoul	2.4 ± 0.1	2.3 ± 0.1
Venice	2.23 ± 0.04	2.2 ± 0.1
Vienna	2.20 ± 0.04	2.20 ± 0.03
Walnut Creek	2.6 ± 0.1	2.4 ± 0.1
Washington	2.25 ± 0.04	2.24 ± 0.03

Table S1. The market competition dimension m_d for 19 different cities from all over the world. The $N(r)^{1/m_d}$ fit column shows the results obtained by fitting how the maximum of the equilibrium average price $\langle p^* \rangle$ scales with N, the number of nodes in the network. As a consistency check we report in N_{max}^{1/m_d} fit column the values of the market dimension obtained simply from $\langle p^* \rangle = N_{max}^{1/m_d}$, where for each city $N_{max} = N(r = 7)$ is the maximum order of the sampled graph, corresponding to a neighbourhood of range $r = 7$ of the node with the highest closeness centrality.

position n of one of the two sellers. For example, we can focus on seller α, and hence $\overline{d}_{n_\alpha}$ is the average transportation cost (measured in units of distance) of the buyers toward the closest seller, as a function of the node occupied by seller α. That is:

$$\overline{d}_{n_\alpha} = \sum_{n_\beta} \sum_j \left[d_{n_\alpha n_j} \frac{\delta(min(d_{n_\alpha n_j}, d_{n_\beta}) - d_{n_\alpha n_j})}{\sum_j \delta(min(d_{n_\alpha n_j}, d_{n_\beta n_j}) - d_{n_\alpha n_j})} + d_{n_\beta n_j} \frac{\delta(min(d_{n_\beta n_j}, d_{n_\alpha n_j}) - d_{n_\beta n_j})}{\sum_j \delta(min(d_{n_\beta n_j}, d_{n_\alpha n_j}) - d_{n_\beta n_j})} \right] \quad [50]$$

where $\delta(\cdot)$ is the Kronecker's delta. Fig. S7 reports the values of HC for BR price update and $\overline{d}$ as a function of the node label n for a chain of $N = 50$ nodes, where as usual the nodes are labelled using integer numbers in increasing order. Both HC and $\overline{d}$ are normalized between 0 and 1. We observe that the two nodes $n = 13$, $n = 38$ (i.e. corresponding respectively to

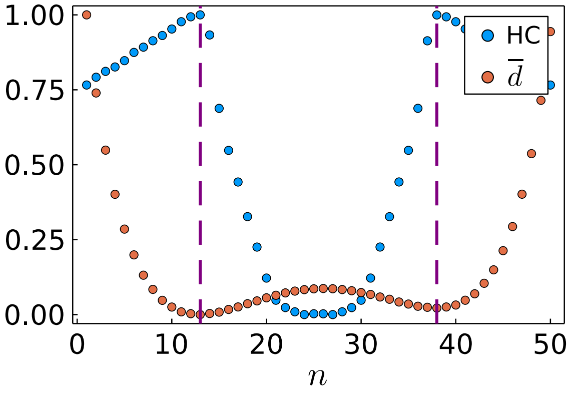

Fig. S7. HC for BR price update compared to $\overline{d}$, for the nodes of a chain of cardinality $N = 50$. The two dashed lines indicate the two nodes $n = 13$ and $n = 38$ which at the same time maximize HC and minimize $\overline{d}$.

$n/N \approx 0.25, 0.75$) maximize HC while at the same time minimize $\overline{d}$. We recall that from the definition of HC (see main text) it follows that nodes with the highest HC are the best positions that a seller can occupy to maximize its earning, when the seller

has no information on the position of the other seller. This means that on a chain market, the positions which guarantee the average highest payoff to a seller are also the ones minimizing the transportation cost of the buyers.

References

1. MJ Osborne, A Rubinstein, *A Course in Game Theory.* (The MIT Press, Cambridge, USA), (1994).
2. V Latora, V Nicosia, G Russo, *Complex Networks: Principles, Methods and Applications.* (Cambridge University Press), (2017).
3. MEJ Newman, *Networks.* (Oxford University Press), (2018).
4. P Crucitti, V Latora, S Porta, Centrality measures in spatial networks of urban streets. *Phys. Rev. E* **73**, 036125 (2006).
5. A Cardillo, S Scellato, V Latora, S Porta, Structural properties of planar graphs of urban street patterns. *Phys. Rev. E* **73**, 066107 (2006).
